# Lipopolysaccharide and Tumor Necrosis Factor Regulate Parkin Expression via Nuclear Factor-Kappa B

**DOI:** 10.1371/journal.pone.0023660

**Published:** 2011-08-17

**Authors:** Thi A. Tran, Andrew D. Nguyen, Jianjun Chang, Matthew S. Goldberg, Jae-Kyung Lee, Malú G. Tansey

**Affiliations:** 1 Departments of Physiology, The University of Texas Southwestern Medical Center, Dallas, Texas, United States of America; 2 Department of Physiology, Emory University School of Medicine, Atlanta, Georgia, United States of America; 3 Department of Neurology, The University of Texas Southwestern Medical Center, Dallas, Texas, United States of America; 4 Department of Psychiatry, The University of Texas Southwestern Medical Center, Dallas, Texas, United States of America; National Institute on Aging Intramural Research Program, United States of America

## Abstract

Inflammation and oxidative stress have been implicated in the pathophysiology of Parkinson's disease (PD) and inhibition of microglial activation attenuates degeneration of dopaminergic (DA) neurons in animal models of PD. Loss-of-function mutations in the *parkin* gene, which encodes an E3 ubiquitin ligase, cause autosomal recessive parkinsonism. While most studies on Parkin have focused on its function in neurons, here we demonstrate that Parkin mRNA and protein is detectable in brain-resident microglia and peripheral macrophages. Using pharmacologic and genetic approaches, we found that Parkin levels are regulated by inflammatory signaling. Specifically, exposure to LPS or Tumor Necrosis Factor (TNF) induced a transient and dose-dependent decrease in Parkin mRNA and protein in microglia, macrophages and neuronal cells blockable by inhibitors of Nuclear Factor-Kappa B (NF-κB) signaling and not observed in MyD88-null cells. Moreover, using luciferase reporter assays, we identified an NF-κB response element in the mouse *parkin* promoter responsible for mediating the transcriptional repression, which was abrogated when the consensus sequence was mutated. Functionally, activated macrophages from Parkin-null mice displayed increased levels of TNF, IL-1β, and iNOS mRNA compared to wild type macrophages but no difference in levels of Nrf2, HO-1, or NQO1. One implication of our findings is that chronic inflammatory conditions may reduce Parkin levels and phenocopy *parkin* loss-of-function mutations, thereby increasing the vulnerability for degeneration of the nigrostriatal pathway and development of PD.

## Introduction

Parkinson's disease (PD) is a progressive, neurodegenerative disorder affecting approximately 1–2% of the adult population over 65 years of age [1]. The majority of PD cases are thought be sporadic, meaning the disease arises in individuals without genetic susceptibility for PD [2]. Several lines of evidence implicate neuroinflammation as a contributing factor in PD pathogenesis [3], [4], [5], [6]. Notably, PET imaging studies of PD patients show increased neuroinflammation as evidenced by microglial activation in brain regions most affected by PD compared to healthy, age-matched controls [7]. Neuroinflammation is largely characterized by activation of microglia [8]. These monocyte-derived brain resident macrophages are central players in innate immune responses in the brain. Environmental toxins, genetic mutations, traumatic brain injury, and infection are among the factors known to activate microglia [9], [10].

Mutations in the *parkin* gene (PARK2), which encode for an E3 ubiquitin ligase, are the leading cause of early-onset, autosomal recessive parkinsonism [11], [12], [13]. Parkin levels in neurons are associated with protection from cellular stress and cell cycle regulation. Treatment of SH-SY5Y cells with rotenone, glutamate or kainic acid induced increases in Parkin protein and mRNA within 3 to 8 hours of exposure [14]. Additionally, Parkin expression was recently reported to increase with mitochondrial or ER stress as mediated by ATF4 [15]. In regards to cell cycle regulation, increased levels of Parkin were associated with cell cycle arrest in neuroblastoma cells, which is transcriptionally mediated by N-myc [16]. These findings demonstrate that Parkin levels are transcriptionally regulated in neurons to control development and cellular stress. However, whether Parkin expression is regulated in microglia and whether levels are affected by cellular functioning is still unknown.

We recently demonstrated that the nigrostriatal pathway of Parkin-null mice displayed increased vulnerability to inflammation-induced degeneration when exposed to chronic systemic lipopolysaccharide (LPS) [17]. This finding demonstrated that inflammatory activation not only in the central nervous system (CNS) but also in the periphery may influence neuronal survival in the CNS. Therefore, we posit that gene-environment interactions between inflammatory pathways in glial cells, and gene products such as Parkin, are critical for protection against cellular stress which may contribute to the development of PD. In support of this idea, we report that Parkin levels in microglia and macrophages are dynamically regulated at the transcriptional level in an NF-κB-dependent manner.

## Materials and Methods

### Animal Studies

Experimental procedures involving use of animal tissue were performed in accordance with the NIH Guidelines for Animal Care and Use and approved by the Institutional Animal Care and Use Committee at The University of Texas Southwestern Medical Center in Dallas, Texas (Protocol No. 0949-06-11-1 and 0949-04-05-1) and Emory University School of Medicine in Atlanta, Georgia (Protocol No. 208-2009). Animals were housed in climate-controlled pathogen-free facilities staffed with certified veterinarians, maintained on a twelve-hour lights-on/off cycle, and allowed food and water *ad libitum*. Parkin-null mice were generated as previously described [18] and have been backcrossed to strain C57BL/6 for over ten generations. TNF-null (B6;129S6-Tnftm1Gkl/J; stock number 003008) mice on a C57BL/6 background strain along with wild type mice as non-transgenic controls were obtained from Jackson Labs (Bar Harbor, ME).

### Reagents

Lipopolysaccharide (LPS) *E.coli* strain 0111:B4 (Catalog # L4391) was purchased from Sigma-Aldrich (St. Louis, MO). SN50 NF-κB peptide (Cat#481480) was purchased from Calbiochem. Tumor necrosis alpha (TNF) was purchased from R&D Systems (Minneapolis, MN). CDDO-Im was a kind gift from Dr. Michael Sporn (Dartmouth University).

### Cell Culture

The murine BV2 microglia cell line was generated by Dr. Bistoni and colleagues by infecting primary microglial cell cultures with the v-raf/v-myc oncogene carrying retrovirus J2 [19]. BV2 microglial cells were cultured in DMEM/F12 (Sigma-Aldrich) supplemented with 5% heat-inactivated fetal bovine serum (FBS), 1% penicillin/streptomycin, and 1% L-glutamine all purchased from Sigma-Aldrich. Cells were serially passaged when they reached 70% confluence. The murine clonal hybrid cell line MN9D was developed by A. Heller and colleagues by somatic cell fusion of rostral mesencephalic tegmentum from E14 mice and the murine neuroblastoma cell line N18TG2 [20]. MN9D cells were grown in DMEM (Sigma-Aldrich) supplemented with 10% Fetal Clone III (Hyclone) and 1% penicillin/streptomycin. Cells were serially passaged when they reached 70% confluence. To induce terminal neuron-like differentiation of MN9D cells, cells were incubated with 5 mM valproic acid (Sigma-Aldrich) in N2 (Invitrogen)-supplemented serum-free DMEM (Sigma-Aldrich) for 3 days.

### Culture of Primary Microglia

Primary microglia were harvested from postnatal day 2–4 (P2–P4) wild type C57BL/6 or Parkin-null mouse pups using previously published protocols [21]. Briefly, brain tissue was removed, finely minced with a razor, incubated in 0.25% trypsin (Sigma-Aldrich) containing 0.5% v/v DNaseI (Invitrogen) for 20 minutes at 37°C. After neutralization with serum-containing DMEM/F12 growth medium, cells were centrifuged, passed through a 40 µM filter (BD Falcon), and plated in cell culture flasks. Cells were fed every 3–4 days with fresh media (DMEM/F12 supplemented with 20% heat-inactivated FBS (Sigma-Aldrich), 1% penicillin-streptomycin, and 1% L-glutamine (Sigma-Aldrich)). After 14–18 days *in vitro*, microglia were isolated from cultures by mechanical agitation (150 rpm, 40 min at 37°C). The cultures were checked for purity and found to contain >95% microglia as determined by cell-type specific expression of CD68 and less than 5% astrocytes as determined by GFAP immunoreactivity.

### Culture of Primary Macrophages

Murine peritoneal macrophages were obtained by eliciting an acute peripheral inflammatory reaction with an intraperitoneal (i.p.) injection of thioglycolate [22]. Briefly, adult mice were given an i.p. injection of 3% Brewer's yeast thioglycolate in normal saline. Three days later peritoneal exudates were recovered, pelleted and resuspended in culture media (high-glucose DMEM supplemented with 10% FBS from Atlanta Biologicals (Norcross, GA), 1% penicillin/streptomycin, and 1% L-glutamine (Sigma-Aldrich). Six hours after the initial plating, cells were washed twice with PBS to remove non-adherent cells and growth medium was replenished to the homogeneous population of adherent macrophages.

### Immunoblot Analysis

Cells were treated as indicated and washed once with PBS before harvesting with 2× Laemmli buffer. Total cell lysates were stored at −80°C until use. Cell lysate proteins were resolved on 10% SDS-PAGE gels (Bio-Rad, Hercules, CA) and transferred onto PDVF membranes (Millipore, Billerica, MA). Immunoblotting was performed using anti-Parkin (PARK8) (Covance, Princeton, NJ) and anti-alpha tubulin (Calbiochem, Gibbstown, NJ) antibodies. Membranes were stripped with 0.2 M glycine, 1% SDS and 0.1% Tween-20, pH 2.2 and re-probed as necessary.

### Quantitative Real-Time Polymerase Chain Reaction (QPCR)

QPCR was performed as previously described [23]. Briefly, total RNA was isolated from cells in culture using Tri-Reagent (Molecular Research Center Inc., Cincinnati, OH) or the RNeasy isolation kit (Qiagen, Valencia, CA), treated with DNaseI, and reverse transcribed using Superscript II RNase H- reverse transcriptase (Invitrogen). QPCR was performed using SYBR Green in 384-well format using an ABI Prism 7900HT Fast Detection System (Applied Biosystems, Foster City, CA). Oligonucleotide primers for QPCR were obtained from Integrated DNA Technologies (Coralville, IA). All mouse primer sequences (available upon request) were validated and used for gene amplification. Levels of mRNA expression were normalized to those of the mouse house-keeping genes cyclophilin B, GAPDH and/or β-actin. Values represent the mean value of triplicate samples +/− SEM. Data are representative of at least two independent experiments.

### Generation of Parkin Reporter Plasmids

Sequences containing the 5′-flanking region of the mouse *parkin* gene were amplified by PCR with the Phusion DNA polymerase kit (New England Biolabs, Ipswich, MA). Mouse genomic DNA isolated from a C57BL/6 mouse was used as the template. The forward and reverse primers used for amplification of Parkin #1 (see Fig. 4A) are GAGATGGTGTGACAAGGGGAATGAGAAG and CCCCTGTCGCTTAGCAACCACTTC, respectively. The forward and reverse primers used for amplification of Parkin #2 are ATGCTGCCTTTGAGTTTTATGCAAAGG and CCCCTGTCGCTTAGCAACCACTTC. The PCR products were gel purified, subjected to restriction digestion with KpnI and NheI, and subcloned into the multiple cloning site of the promoterless pGL4.10 vector from Promega (Sunnyvale, CA) upstream of a synthetic luciferase coding sequence. Site-directed mutagenesis of the candidate NF-κB binding site (GGGGCATCC) was performed using the QuikChange XL kit (Stratagene, Boston, MA) using the pGL4-Parkin #1 plasmid as the template. The forward and reverse primers used were CCACCATTTCTTTCTGCCGCTAGCACATGCTGCCTTTGAG and CTCAAAGGCAGCATGTGCTAGCGGCAGAAAGAAATGGTGG, with the mutated nucleotides underlined. The pGL4-tk-Renilla luciferase vector, in which Renilla luciferase is constitutively expressed under the control of the thymidine kinase (tk) promoter, was obtained from Promega (pGL4.74). The integrity of each plasmid was confirmed by DNA sequencing.

### Transfection of Reporter Plasmids and Luciferase Assays

MN9D cells were plated in 24-well plates at a density of 8×10^4^ cells per well. The following day, the cells of each well were transfected with 450 ng of the indicated reporter plasmid and 50 ng of the pGL4-tk-Renilla Luciferase plasmid using 1.5 µL of FuGene6 (Roche Applied Science, Indianapolis, IN). After 4–6 hours, cells were switched to serum-free media. The following day, cells were treated with either PBS (vehicle) or 10 ng/mL TNF for 18 hours. At the end of the treatment period, firefly and Renilla luciferase activities were measured using the Dual-Glo Luciferase Assay System (Promega) according to the manufacturer's instructions. The amount of firefly luciferase activity of the transfected cells was normalized to Renilla luciferase activity. Data are expressed as the relative luciferase activity compared to that of vehicle-treated cells that were transfected with the corresponding reporter plasmid (which is set at 100% for each reporter plasmid). TNF treatment did not affect the Renilla luciferase activity of the internal control plasmid or the low level firefly luciferase activity of the empty pGL4 plasmid.

### Multiplexed High-Sensitivity Immunoassays

Conditioned media from mixed glia cultures from P2-P4 mouse pups were collected to measure the protein concentration of seven inflammatory mediators (IFN-γ, IL-1β, IL-6, IL-10, IL-12, KC/CXCL1, and TNF) using a high-sensitivity multiplexed immunoassay per the manufacturer's instructions (Meso-Scale Discovery) as published previously [24].

## Results

### Parkin mRNA and protein levels in brain microglia and peripheral macrophages are decreased by LPS and TNF

Previous work from our group demonstrated that Parkin-null mice displayed increased vulnerability to degeneration of DA neurons in the ventral midbrain substantia nigra pars compacta (SNpc) induced by chronic systemic inflammation [17]. While the mechanism(s) by which Parkin protects the nigrostriatal pathway against chronic inflammatory stress remain unknown, these observations support the existence of important interactions between Parkin and inflammatory factor signaling during innate immune system activation.

**Figure 1:**
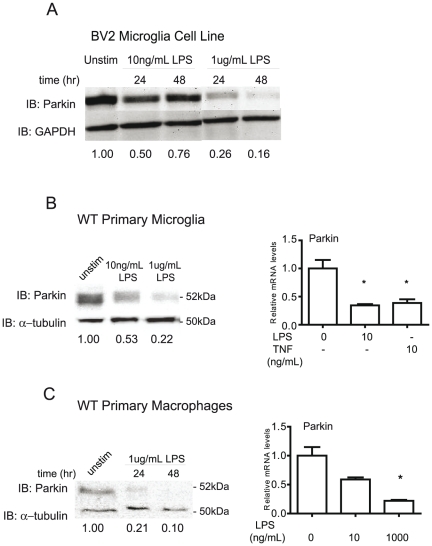
Parkin protein and mRNA expression in brain microglia and peripheral macrophages is regulated by LPS and TNF. *A,* Parkin levels in BV2 microglia cells (plated in 6-well plates at 0.5×10^6^ cells/well) were treated with LPS as indicated. *B,* Parkin levels in primary microglia (plated in 24-well plates at 0.1×10^6^ cells/well) were treated with LPS or TNF as indicated for 24 hr. Protein levels measured by immunoblotting are shown on the left; Parkin mRNA levels as measured by real-time PCR (QPCR) are shown on the right. *C,* Parkin levels in primary peritoneal macrophages (plated in 6-well plates at 0.8×10^6^ cells/well) were treated with LPS or TNF as indicated. The values indicate relative levels of Parkin protein compared with housekeeping gene GAPDH or α-tubulin. QPCR analysis was performed by two-way ANOVA followed by Tukey's post hoc test, * p<0.05 compared to the unstimulated sample. Data are representative of two to three independent dissections and treatments.

First, we investigated the extent to which Parkin is expressed in microglia and macrophages, cells involved in innate immunity. We found that Parkin mRNA and protein are detectable by real-time PCR and immunoblotting techniques respectively, in BV2 microglia, primary microglia and peritoneal macrophages (**Figure 1**). Next, we investigated the extent to which Parkin levels are regulated by inflammatory factors. Based on reports that rotenone, glutamate and kainic acid exposure increased Parkin mRNA and protein levels in neuronal cells [14], we expected that inflammatory stimuli would also increase Parkin expression in microglia and macrophages. Surprisingly, immunoblot analyses of Parkin protein showed that treatment of the BV2 microglial cell line with 10 ng/mL LPS decreased Parkin levels by 50% from resting levels after 24 hours and by 24% after 48 hours; stimulation with 1 ug/mL LPS decreased Parkin levels by 74% after 24 hours and by 85% after 48 hours (**Figure 1A**). We confirmed these findings in primary microglia from wild type mice (**Figure 1B**). To extend these findings, we measured Parkin mRNA by real-time PCR and found that Parkin mRNA levels were also repressed by 10 ng/mL LPS or 10 ng/mL TNF. Lastly, we observed similar results in LPS-treated primary macrophages, the systemic cellular counterpart of brain microglia (**Figure 1C**). These data suggest that inflammatory mediators may negatively regulate transcription of Parkin mRNA and the transcriptional repression accounts for decreased Parkin protein levels.

**Figure 2:**
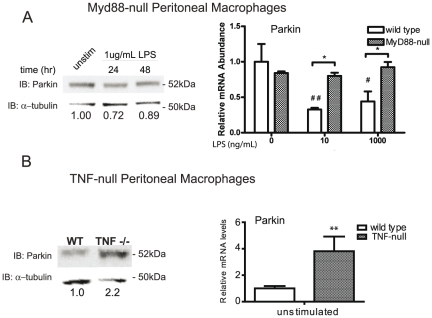
Parkin downregulation by LPS requires intact TLR4 signal transduction. *A,* Primary peritoneal macrophages isolated from adult wild type or MyD88-null mice were plated in 6-well plates at 0.8×10^6^ cells/well) and treated as indicated. Protein levels measured by immunoblot are shown on the left; Parkin mRNA levels measured by real-time PCR (QPCR) are shown on the right. *B,* Parkin levels in naïve (untreated) macrophages harvested from TNF-null mice. The values indicate relative levels of Parkin protein compared with that of the housekeeping gene α-tubulin. QPCR analysis was performed by two-way ANOVA followed by Tukey's post hoc test, ** p<0.01 compared to wild type sample and ## p<0.01 compared to the unstimulated sample. Data are representative of two independent dissections and treatments.

Next we investigated specificity of the LPS-induced downregulation by testing for the necessity of proteins involved in LPS signal transduction. The LPS receptor Toll-like receptor-4 (TLR4) and its co-receptor MD-2 must recruit the adaptor protein MyD88 to the cell surface to propagate inflammatory signaling [25]. We harvested macrophages from MyD88-null mice, which lack LPS-induced cytokine production [26]. Unlike peritoneal macrophages harvested from wild type mice, macrophages isolated from MyD88-null mice do not display downregulation of Parkin protein or mRNA when stimulated with LPS (**Figure 2A**). Tumor necrosis factor (TNF) is a potent inflammatory cytokine that elicits classic activation responses in a number of immune cell types through NF-κB-dependent signaling. To further investigate the extent to which Parkin expression is acutely regulated by inflammatory factors, we measured Parkin expression in peritoneal macrophages harvested from TNF-null mice. We observed greater than two-fold increases in basal levels of Parkin protein and mRNA levels in macrophages harvested from TNF-null mice compared to those harvested from wild type mice (**Figure 2B**). Together, these findings indicate that LPS and TNF negatively regulate Parkin expression in microglia and macrophages.

### NF-κB pathway activation mediates downregulation of Parkin expression in microglia by LPS

**Figure 3:**
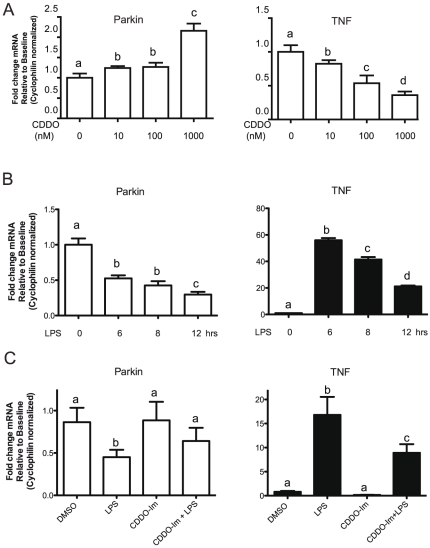
TNF and LPS induce downregulation of Parkin in an NF-κB-dependent manner. *A,* BV2 cells were plated into 6-well plates at a density of 0.5×10^6^ cells per well. Parkin and TNF mRNA expression was measured in BV2 cells following an overnight incubation with only the synthetic triterpenoid CDDO-Im at the concentrations indicated or in the vehicle DMSO. *B,* Parkin and TNF mRNA expression was measured in BV2 cells after stimulation with LPS (1 ug/mL) for the times indicated. *C,* Inhibition of NF-κB pathway activation in BV2 microglia cells with the synthetic triterpenoid CDDO-Im (1 µM in DMSO) or its vehicle during LPS stimulation ablated LPS-induced repression of Parkin mRNA and attenuated LPS induction of TNF mRNA (positive control). One way ANOVA followed by Tukey's post hoc test, columns with different letters are significantly different from each other, p<0.05.

Nuclear Factor Kappa-B (NF-κB) is a signaling pathway that regulates many important processes in innate immune cells [27]. In resting microglia, NF-κB is bound to IκB and remains primarily in the cytoplasmic compartment; in activated cells IκB is phosphorylated by IκB kinase (IKK) and subsequently degraded which releases NF-κB, allowing it to translocate to the nucleus to activate transcription of target genes [28]. CDDO-Imidazole (CDDO-Im), a synthetic triterpenoid derivative of oleanolic acid blocks activation of NF-κB [29], [30], [31]. Therefore, we employed CDDO-Im to test the hypothesis that Parkin expression is mediated by NF-κB signaling. First, we measured the mRNA levels of Parkin and TNF, a classic NF-κB target gene, in the presence of various concentrations of CDDO-Im in resting BV2 cells. We found that CDDO-Im induced a dose-dependent increase in Parkin mRNA and a concomitant decrease in TNF mRNA as measured by real-time PCR (**Figure 3A**). Next, we measured the kinetics of LPS-induced changes in Parkin and TNF mRNA in BV2 cells. We found that LPS induced a rapid decrease in Parkin mRNA (70% decrease from baseline at 12 hrs post stimulation) and the expected transient increase in TNF mRNA) (**Figure 3B**). To test the effects of NF-κB inhibition during LPS stimulation, we pre-incubated BV2 microglia cells in DMSO vehicle or 1 µM of CDDO-Im for 1 hour then measured changes in Parkin and TNF mRNA levels after exposure to 12 hrs of 1 ug/mL LPS. Inhibition of NF-κB by pre-treatment with CDDO-Im resulted in increased Parkin mRNA levels; CDDO-Im decreased TNF mRNA expression as expected (**Figure 3C**). Similar results were observed in BV2 cells treated with either N-α-tosyl-L-lysine chloromethyl ketone (TLCK), a proteasome inhibitor shown to block NF-κB activation by suppressing IκB degradation in RAW 264.7 macrophages [32] (data not shown), or with SN50, a cell-permeable peptide that inhibits translocation of the NF-κB active complex into the nucleus [33] (data not shown). In summary, genetic and pharmacological data support the involvement of NF-κB activation in downregulation of microglial Parkin mRNA in response to LPS.

### The mouse *parkin* gene promoter contains an NF-κB regulatory site

**Figure 4:**
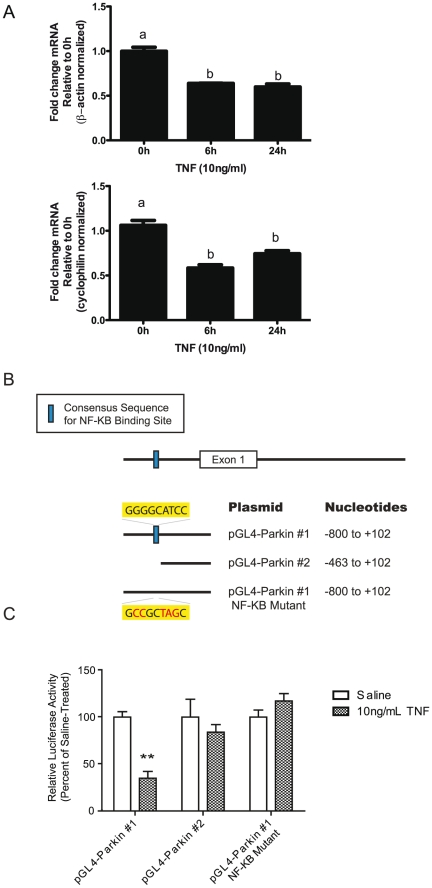
The mouse *parkin* promoter contains a putative NF-κB binding site. *A,* TNF induces downregulation of Parkin mRNA in MN9D dopaminergic neuroblastomacells. Cells were plated at a density of 3×10^5^ cells per well in 6-well plates (3 wells per condition). Parkin mRNA expression was measured in MN9D cells after stimulation with TNF (10 ng/mL) for the times indicated. One way ANOVA followed by Tukey's post hoc test, columns with different letters are significantly different from each other, p<0.05. *B,* Schematic of the luciferase reporter plasmids driven by the mouse *parkin* promoter, either including (#1) or excluding (#2) the putative NF-κB binding site. *C,* MN9D dopaminergic cells were plated at a density of 8×10^4^ cell/well in 24-well plates (3 wells per condition) and transfected with the pGL4-Parkin reporter plasmids (#1, #2 or #1 mutant) and Renilla luciferase plasmid. Firefly and Renilla luciferase activities were measured in a luminometer after an overnight treatment of 10 ng/mL TNF. Firefly luciferase activity was normalized to internal control Renilla luciferase activity. Data are expressed as the relative luciferase activity compared to that of vehicle-treated cells that were transfected with the corresponding reporter plasmid (which is set at 100% for each reporter plasmid). ANOVA followed by Tukey's post hoc test, * p<0.05. Data are representative of two independent transfections.

We hypothesized that if NF-κB was involved in the regulation of Parkin expression, stimulation of neuronal cells with TNF should also activate NF-κB signaling and result in downregulation of Parkin mRNA expression. To test this directly, we treated neurally differentiated ventral mesencephalon MN9D neuroblastoma cells with 10 ng/mL TNF and harvested the cells for RNA extraction after 6, 12 or 24 hours of stimulation. In agreement with results obtained in TNF-treated microglia and macrophages, QPCR analysis revealed that TNF treatment induced downregulation of Parkin mRNA in primary dopaminergic neuroblastoma cells (**Figure 4A**). Next, we determined whether NF-κB was directly regulating Parkin expression at the promoter level. Examination of the mouse *parkin* promoter revealed the presence of sequence motifs found in NF-κB target genes. The sequence 5′-GGGRNNYYCC-3′ where R is A or G and Y is C or T has been proposed to be an NF-κB target gene consensus sequence based on alignment of established NF-κB binding sites [34]. Sequence analysis of the mouse *parkin* gene, including 3.2 kb upstream of the transcriptional start site, revealed the presence of a sequence that closely resembles this consensus sequence. We performed reporter assays to determine whether this site on the *parkin* promoter was a regulatory element necessary for inhibiting Parkin transcription. To this end we generated two reporter plasmids in which the mouse *parkin* gene promoter was cloned upstream of a luciferase-coding sequence in the promoterless pGL4 vector, either including (pGL4-Parkin #1) or excluding (pGL4-Parkin #2) the site of interest, (**Figure 4B**). To ensure high transfection efficiency, we transfected these Luc-reporter plasmids into MN9D dopaminergic neuroblastoma cells along with a plasmid that constitutively expresses Renilla luciferase as an internal control. The transfected cells were subsequently treated with saline or 10 ng/mL TNF for 18 hours after which firefly and Renilla luciferase activities were measured in a luminometer. We found that TNF treatment resulted in a 65% decrease in luciferase activity in MN9D cells transfected with the pGL4-Parkin #1 plasmid which contains the putative NF-κB binding site (**Figure 4C**). In contrast, no change in luciferase activity was observed in response to TNF treatment in MN9D cells transfected with the pGL4-Parkin #2 plasmid (which lacks the putative NF-κB binding site in the mouse *parkin* promoter). Site directed mutagenesis of this sequence in the *parkin* gene promoter ablated the TNF-dependent repression of luciferase activity driven by the *parkin* promoter (pGL4-Parkin #1 NF-κB Mutant), supporting our idea that Parkin expression is regulated at the transcriptional level by NF-κB,. These reporter assay experiments reveal that the promoter of the mouse *parkin* gene contains a functional NF-κB binding site. Together with pharmacological studies, our findings strongly suggest that Parkin expression is repressed at the transcriptional level by NF-κB pathway activation in cells treated with LPS or TNF.

### Parkin expression levels in immune cells affect inflammatory gene expression

**Figure 5:**
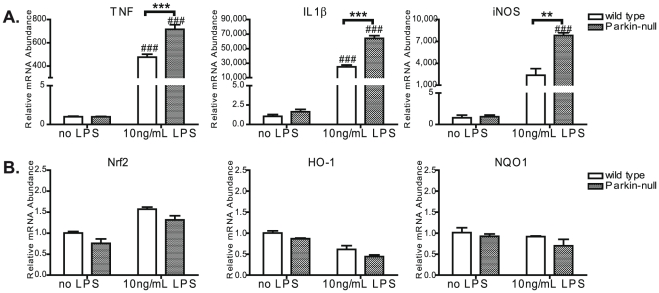
Parkin expression levels in macrophages affect inflammatory gene expression. Peritoneal macrophages were isolated from 18-month old wild type and Parkin-null mice. Cells were plated and treated with 10 ng/mL LPS for 4 hours after which RNA was collected and processed for QPCR analysis to measure levels of inflammatory (**A**) and antioxidant genes (**B**). Two-way ANOVA with Bonferroni's post doc analysis, #p<0.05 and ###p<0.001 compared to no LPS treatment, while **p<0.01 and ***p<0.001 compared to wild type.

To explore the extent to which repression of Parkin expression by inflammatory stimuli alters cellular effector functions, we examined the expression levels of inflammatory and antioxidant markers in resting or activated peritoneal macrophages from wild type and Parkin-null mice. Isolated peritoneal macrophages were treated with 10 ng/mL LPS for 4 hours prior to RNA harvest for real time PCR analyses. Activated macrophages from Parkin-null mice expressed increased levels of TNF, IL-1β, and iNOS mRNA compared to wild type macrophages (**Figure 5A**), but no difference in levels of Nrf2, HO-1, and NQO1 (**Figure 5B**). We detected no change in expression of TLR4 or TLR2 (data not shown). These data suggest a novel role in which Parkin may limit expression of inflammatory mediators in resting and activated innate immune cells. Thus, downregulation of Parkin expression by NF- κB in LPS or TNF-treated cells may be necessary to elicit the typical increasedexpression of a subset of inflammatory genes in activated cells; consistent with this idea is the exacerbated inflammatory response of LPS treated macrophages from Parkin-null mice.

## Discussion

**Figure 6:**
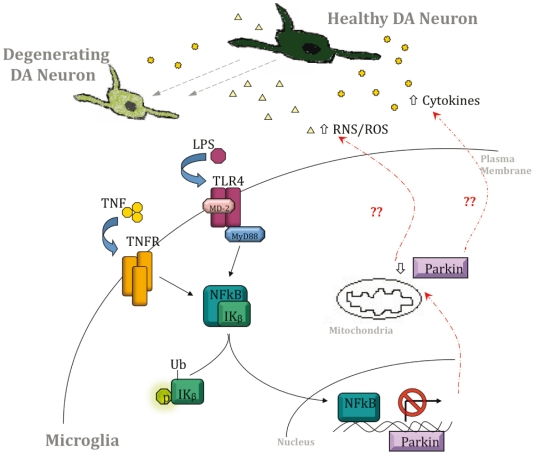
Proposed model of LPS-induced and NF-κB-dependent Parkin downregulation. LPS and TNF stimulate microglia and macrophages by binding TLR4 and TNF receptors respectively to propagate intracellular signaling through adapter proteins and the transcription factor NF-κB. Once activated, NF-κB translocates to the nucleus where it binds to the *parkin* promoter to repress transcriptional activity. We speculate that downregulation of Parkin during periods of chronic inflammatory stress may generate a feed-forward system that enhances inflammation-related toxicity in the microenvironment surrounding DA neurons thereby promoting progressive neuronal degeneration.

Based on the results from genetic, biochemical and pharmacological studies presented here, we propose a working model (**Figure 6**) by which LPS and TNF stimulate microglia and macrophages by binding TLR4 and TNF receptors, respectively to result in activation of NF-κB-dependent gene transcription in activated cells. Activation of this important pathway transiently decreases Parkin mRNA and protein levels in microglia and macrophages, suggesting that dynamic regulation of Parkin levels may subserve an important role of Parkin in these cell types that may include limiting expression of genes involved in inflammatory responses, as suggested by our QPCR analyses. Microglia and macrophages respond to external stimuli in order to perform their immune surveillance role in the central nervous system (CNS) and peripheral circulation, respectively and they activate the NF-κB signaling pathway in response to LPS and TNF treatment [27], [28]. Although NF-κB activates the expression of many target genes, it has also been reported to repress gene transcription, including that of the rat androgen receptor [35]. Moreover, the *Drosophila melanogaster* homolog of NF-κB/Rel (*dorsal*), positively regulates some genes while negatively regulating others during embryonic development [36]. *dorsal* is reported to act as a repressor when it associates with a neighboring co-repressor. The LPS-induced repression of Parkin expression may occur through a similar mechanism involving a co-repressor. The identification of a putative NF-κB site in the mouse *parkin* promoter is a novel observation, and to our knowledge this is the first demonstration that Parkin transcription is repressed by inflammatory stimuli in microglia, macrophages and neurally differentiated dopaminergic neuroblastoma cells.

Importantly, our finding that LPS and TNF-dependent NF-κB signaling, which serves to activate microglia, also represses Parkin levels suggests a complex interaction between neuroinflammatory signaling and Parkin. One possible result of this interaction is that a transient decrease in Parkin levels may be necessary for normal activation and proliferation of microglia and macrophages following LPS or TNF stimulation. In support of this idea, Parkin expression was found to be transcriptionally repressed in dividing cells [16]. Although this instance of repression was mediated by N-myc, this example demonstrates a cellular mechanism of Parkin downregulation during cellular proliferation. A similar example of regulation through repression occurs in apoptotic signaling cascades when specific members of the inhibitor of apoptosis protein (IAP) family are sequestered or destroyed in order for apoptotic signaling to proceed [37], [38]. While it is possible that dynamic changes in Parkin levels subserve an important and as yet undefined role of Parkin in microglial activation, complete loss of Parkin may contribute to dysregulated microglial responses in the CNS. In support of this idea, our data that LPS-treated Parkin-null macrophages displayed heightened inflammation-related gene expression suggests potentially important functional consequences as a result of complete loss of Parkin in monocytes. Conversely, microglia or macrophages with atypically high levels of Parkin (such as those from TNF-null mice) displayed blunted activation responses, in agreement with studies that show macrophages from TNF-null mice display weakened activation responses when challenged with oxidative stress [39], [40]. Lastly, Parkin-null mice have been reported to display increased microglial proliferation and activation in the CNS [41] and mitochondrial alterations in glial cells have been associated with an inability to provide trophic support to neuronal cultures [42].

The functional consequences of acute and chronic downregulation of Parkin levels in microglia and macrophages and its impact on DA neurons will be addressed in future studies; however, several important predictions can be made on the basis of our findings and our model. O-glycosylated α-synuclein (αSp22) has been shown to be a Parkin substrate [43] and one expected consequence of chronic downregulation of Parkin levels is likely to be accumulation of Parkin substrates. Consistent with this prediction is the observation that the rate of clearance of α-synuclein aggregates in BV2 microglia is significantly reduced after treatment of the cells with LPS [44]. Although the mechanism by which LPS attenuated α-synuclein clearance in activated microglia in those studies was not explored, our findings suggest that downregulation of Parkin levels by LPS-induced NF-κB signaling might in part account for the accumulation of α-synuclein and perhaps other Parkin substrates; this possibility will need to be tested directly. Another important prediction based on our *in vitro* findings is that chronic neuroinflammation and persistent activation of NF-κB in the CNS during CNS infection has the potential to elicit a sustained reduction in Parkin levels, which could also lead to dysregulation of microglia and a toxic microenvironment for DA neurons. Alternatively, repeated exposure to environmental toxins and/or the aging process itself, both of which are associated with chronic neuroinflammation in the CNS, may also result in sustained reductions of Parkin levels in the CNS. All of these environmental triggers could essentially phenocopy the effect of *parkin* loss, thereby increasing the vulnerability to peripheral inflammation-induced nigral dopaminergic pathway degeneration [17] and may predispose an individual to development of PD.

Given that loss of dopamine (DA)-producing neurons in the substantia nigra pars compacta is the neuropathological hallmark of PD, it is understandable that research in the field has focused on analyzing neuronal phenotypes in PD patients and models of PD-like pathology. However, recent studies suggest the exciting possibilities that genes that influence the risk for development of PD may have important roles in glial cells, and are involved in innate immune function that determine how brain immune surveillance cells respond to insult. Nurr1, a transcription factor involved in the development and maintenance of DA neurons [45], was recently reported to be a critical regulator of cytokine expression in microglia and astrocytes. Elegant studies demonstrated that reduction in Nurr1 expression resulted in excess cytokine production (including TNF) that led to death of DA neurons in LPS-treated mice [46]. These unexpected findings underscore two important ideas: the glial microenvironment surrounding DA neurons is a critical determinant of neuronal health and survival; and functions of ubiquitously expressed genes initially thought to be important only in neurons may also have important roles in glial cells and are likely to be dynamically regulated. In addition, genes involved in histocompatibility and immunocompetence have been identified as risk loci for PD in recent genome-wide association studies (GWAS) [47]. Taken together, these exciting findings provide compelling rationale to seek a deeper understanding of how inflammatory signaling and innate immune processes influence the functional outcome of neuron-glia interactions and contribute to or protect against development of PD, the second most common neurodegenerative disease in the U.S.
